# Oxidized Hyaluronic Acid-Based Sponges: A Promising Biomaterial for Oral Mucosa Lesion Application

**DOI:** 10.3390/ijms262110383

**Published:** 2025-10-25

**Authors:** Clara Alicia Muñoz-Trejo, Martha Gabriela Chuc-Gamboa, Juan V. Cauich-Rodríguez, Rossana Faride Vargas-Coronado, Diana María Escobar-García, Amaury Pozos-Guillen, Fernando Javier Aguilar-Pérez, Alicia Leonor Pinzón-Te, Gualberto Antonio Zumbardo-Bacelis

**Affiliations:** 1 Facultad de Odontología, Universidad Autónoma de Yucatán, Calle 61 A #492 A Centro, Mérida C.P. 97000, Mexico; claramunoztrejo31@gmail.com (C.A.M.-T.); dr.faguilarperez@gmail.com (F.J.A.-P.); alicia.pinzon@correo.uady.mx (A.L.P.-T.); 2 Unidad de Materiales, Centro de Investigación Científica de Yucatán, Calle 43 No. 130, Chuburná de Hidalgo, Mérida C.P. 97205, Mexico; ross@cicy.mx; 3 Laboratorio de Ciencias Básicas, Facultad de Estomatología, Universidad Autónoma de San Luis Potosí, Av. Manuel Nava # 2, Zona Universitaria, San Luis Potosí C.P. 78290, Mexico; diana.escobar@uaslp.mx (D.M.E.-G.); apozos@uaslp.mx (A.P.-G.); 4 Department of Chemical Engineering, Laval University, Quebec, QC G1V 0A6, Canada; gualberto-antonio.zumbardo-bacelis.1@ulaval.ca

**Keywords:** biomaterials, chitosan, hyaluronic acid, wound healing

## Abstract

Chitosan (CHT) and hyaluronic acid (HA) are biomaterials with diverse properties. While each has been individually employed for the treatment of oral lesions, there is a need for further evidence regarding their combined properties. This study compares the effects on the properties and biocompatibility of chitosan sponges, CHT crosslinked with oxidized hyaluronic acid (OHA) (oxidized at 1:1 and 1:2 ratios, respectively), and CHT crosslinked with oxidized hyaluronic acid and polyethylene glycol diglycidyl ether (PEGDE). Spectroscopy revealed reduced free amino groups and the amide I/II ratio in CHT sponges crosslinked with OHA. SEM confirmed the porous network morphology with an average pore size ranging from 155 to 213 μm. TGA indicated the scaffolds’ decomposition temperature (Td) increased from 253° to 308°, with the CHT-OHA 1:2 sponge exhibiting the highest thermal stability. Compression testing highlighted that the chitosan sponges crosslinked with AHO and PEGDE at a 1:2 ratio displayed a higher elastic modulus than the other studied scaffolds. The MTS assay confirmed that the fabricated biomaterials were not cytotoxic. This study demonstrates the enhanced properties and biocompatibility of CHT-OHA and CHT-OHA-PEGDE sponges, highlighting their potential for oral lesion treatment.

## 1. Introduction

Oral mucosa lesions (OMLs) are a prevalent pathology in the oral cavity, frequently affecting the general population [1,2]. These ulcers manifest as vesicle–ulcerative lesions with an erythematous base and a yellowish background, progressing through various stages of evolution [3,4,5]. Despite their common occurrence, the exact etiology of canker sores remains unknown and is attributed to multifactorial causes [6]. Many studies suggest a possible genetic basis, alongside predisposing factors such as trauma, stress, certain foods, hormonal imbalances, tobacco use, syndromes, or even malignant disease processes [7]. Additionally, other potential factors like viral or bacterial actions, vitamin deficiencies, and immunological factors have been proposed [8,9,10].

The prevailing treatment for these lesions primarily involves the application of topical agents, with anesthetics and corticosteroids being the preferred choices. This therapeutic approach is further complemented by systemic treatments and mouthwashes [9,10,11]. A significant portion of available treatments relies on synthetic materials, leading to concerns regarding their biodegradability. Consequently, there is a growing inclination toward utilizing biological materials to enhance treatment options [12].

Chitosan, derived from chitin, is a natural polysaccharide composed of 2-acetamido-2-deoxy-D-glucose and 2-amino-2-deoxy-D-glucose units. It exhibits several advantageous properties, including biocompatibility, biodegradability, mucoadhesion, non-toxicity, hemostatic activity, and antibacterial effects [13,14,15]. These features, together with its ability to form films, scaffolds, and gels, make chitosan highly suitable for biomedical applications [16,17]. In dentistry, it acts as an antimicrobial and antibacterial agent that can inhibit the growth of microorganisms responsible for cavities and gingivitis while improving the efficacy of active ingredients in oral formulations [18,19,20]. The Food and Drug Administration (FDA) has approved its use as a wound dressing due to its capacity to accelerate the healing process [14,15].

Sodium hyaluronate is a natural anionic mucoadhesive polysaccharide consisting of alternating repeating units of D-glucuronic acid (GlcA) and N-acetyl-D-glucosamine (GlcNAc), connected through β-(1,4) and β-(1,3) bonds. It plays a crucial role in the extracellular matrix, promoting cell proliferation and the migration of fibroblasts and keratinocytes [21,22,23]. This biopolymer exhibits a broad spectrum of pharmacological activities, including anti-inflammatory, wound healing, tissue-regenerating, immunomodulatory, anticancer, antiproliferative, antidiabetic, anti-aging, skin-reparative, and cosmetic properties [24]. The combination of sodium hyaluronate with chitosan has been shown to improve the mechanical and biocompatibility properties of the resulting materials [25].

Both chitosan and hyaluronate are polysaccharides with similar structures, widely applicable either individually or in combination in various medical fields. They particularly excel in treating osteoarthritis, as well as in ophthalmology, wound healing in the skin, and dentistry [26]. Considering the continued need to improve the efficacy and innovation of oral lesion treatment, this study aims to evaluate the effectiveness of sponges made from chitosan and oxidized hyaluronic acid. Chitosan and hyaluronic acid sponges can help manage canker sores through several mechanisms: they provide a protective barrier, reduce inflammation, promote wound healing, prevent secondary infections, and alleviate pain [27]. This multifaceted approach not only accelerates the healing process but also offers significant symptomatic relief for individuals suffering from canker sores. This investigation provides a comprehensive evaluation of the physicochemical, mechanical, and biological properties of chitosan and oxidized hyaluronic acid sponges as a potential therapeutic alternative for improving the management of oral mucosa lesions.

Despite the recognized biological potential of chitosan and hyaluronic acid, few studies have explored their combined use in a solid sponge specifically designed for oral mucosal injuries, where maintaining mechanical stability and biocompatibility under humid and dynamic conditions is critical. Therefore, this study aims to synthesize and characterize chitosan/oxidized hyaluronic acid (CHT/OHA) sponges and evaluate their physicochemical, mechanical, and biological properties to determine their suitability as scaffolds for oral wound healing.

Our primary hypothesis is that the oxidation of hyaluronic acid (OHA) will allow crosslinking with chitosan, forming a stable, biocompatible, and mechanically reinforced sponge capable of providing an optimal environment for tissue repair in oral mucosal injuries.

## 2. Results and Discussion

### 2.1. Composition by FTIR Spectroscopy

**Figure 1:**
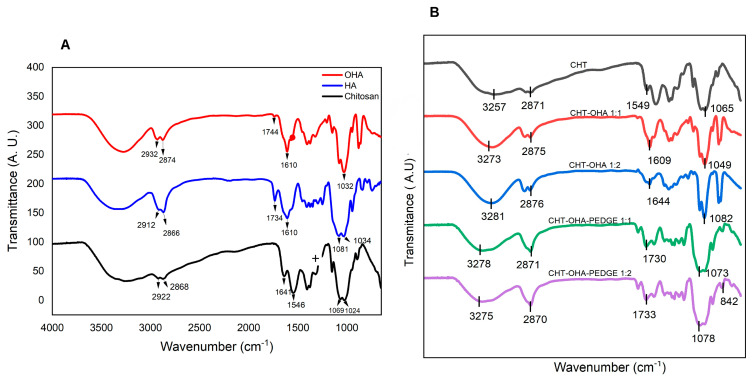
FTIR spectra of (**A**) chitosan (CHT), hyaluronic acid (HA), and oxidized hyaluronic acid (OHA). (**B**) Chitosan/oxidized hyaluronic acid (CHT-OHA) blends and CHT/OHA-PEGDE-crosslinked.

The FTIR spectra of chitosan (CHT), hyaluronic acid (HA), oxidized hyaluronic acid (OHA), and CHT/OHA-PEGDE-crosslinked sponges are presented in Figure 1.

Pristine chitosan (Figure 1A) exhibited a strong absorption band at 3257 cm^−1^ (–OH), with additional peaks at 2922–2868 cm^−1^ attributed to CH stretching of methylene groups. The bands at 1641 cm^−1^ (amide I, C=O/N–H) and 1546 cm^−1^ (amide II, C–H/N–H_2_ bending) correspond to its characteristic amide vibrations, while those at 1408 cm^−1^ (COO^−^) and 1377 cm^−1^ (amide III, NH_2_) are typically used to estimate the degree of acetylation [28]. Other characteristic vibrations of the chitosan backbone were observed at 1152 cm^−1^ (C–O–C), 1069, and 1024 cm^−1^, associated with the pyranose ring and glucosidic linkages [29].

Pristine HA (Figure 1A) exhibited characteristic bands at 3258 cm^−1^ (–OH) and 2919–2886 cm^−1^ (CH_2_ stretching). The absorption near 1734 cm^−1^ corresponds to carbonyl (C=O) vibrations, while 1610 and 1408 cm^−1^ are attributed to the asymmetric and symmetric stretching of carboxylate groups (COO^−^). The band at 1251 cm^−1^ is associated with amide III, corresponding to N–H and C–H bending.

Zhang et al. noted that the successful introduction of the aldehyde group into oxidized hyaluronic acid is verified when adjacent hydroxyl groups of pure hyaluronic acid transform into aldehyde groups post-oxidation with sodium periodate. The success of OHA oxidation reported by Zang is evident by the presence of a small band at approximately 1733 cm^−1^, representing the stretching of the C=O bond, characteristic of the aldehyde group [29]. A comparison spectrum between pure HA and OHA revealed a band shift at 1744 cm^−1^, indicating the characteristic peak of aldehydes. However, this peak is small and at times challenging to discern due to the lower degree of oxidation and the formation of hemiacetals [30,31]. In our study, changes were not clearly observed in the carbonyl region, and only a small shift was detected in the C-H stretching region at 2932–2912 cm^−1^ and 2874–2866 cm^−1^.

When chitosan, featuring amino groups, was combined with oxidized hyaluronic acid containing aldehyde groups, the anticipated formation of imine bonds at 1644 cm^−1^ was masked in the broad carbonyl absorption. Conversely, the intensity of the band corresponding to amide II (1546 cm^−1^) decreased upon crosslinking with OHA (1:1), suggesting reduced effectiveness when OHA was employed as a crosslinking agent. Additionally, a red shift of the amide II band in the CHT-OHA 1:2 sponge was observed at 1611 cm^−1^ [32,33,34]. However, in the case of PEGDE-crosslinked samples, bands at 1730 cm^−1^ and 1733 cm^−1^ were detected, indicating either phase separation or the presence of aldehyde groups in CHT-OHA 1:1-PEGDE and CHT-OHA 1:2-PEGDE, respectively. The amide II band at 1564 cm^−1^ and 1567 cm^−1^ experienced a reduction in intensity, suggesting the crosslinking reaction of PEGDE epoxy groups with the hydroxyl/amine groups of chitosan [35,36,37].

### 2.2. Composition by Raman Spectroscopy 

**Figure 2:**
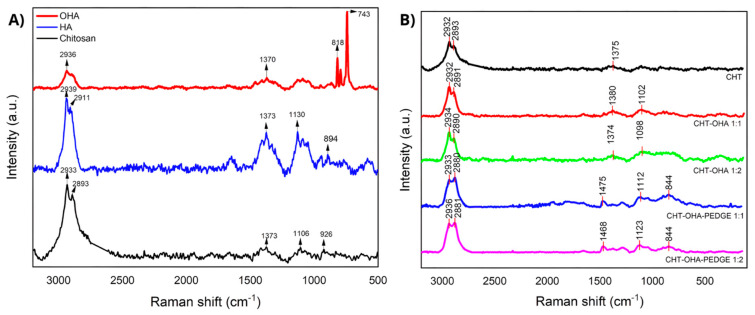
Raman spectra of (**A**) chitosan (CHT), hyaluronic acid (HA), and oxidized hyaluronic acid (OHA). (**B**) Chitosan/oxidized hyaluronic acid (CHT-OHA) blends and chitosan/oxidized hyaluronic acid (CHT-OHA) PEGDE-crosslinked sponges.

Figure 2 illustrates the Raman spectra of chitosan (CHT), chitosan/oxidized hyaluronic acid blends (CHT-OHA 1:1 and 1:2), and their PEGDE-crosslinked counterparts. Pure chitosan (Figure 2A) displayed characteristic peaks at 2933 and 2893 cm^−1^, corresponding to ν(CH_3_) and ν(CH_2_) stretching vibrations, respectively, while pristine HA showed similar bands at 2939 and 2911 cm^−1^.

Pristine HA also exhibited a small band at 1604 cm^−1^, associated with C=C and amide I vibrations. Additional peaks appeared at 1326, 1371 (1373 in this study), and 1454 cm^−1^, corresponding to amide III and asymmetric/symmetric CH bending. The bands at 1135 and 1085 cm^−1^ were attributed to CH and C–OH bending of the acetyl group, respectively, while 894 and 943 cm^−1^ were linked to β-glycosidic linkages and C–C stretching.

Upon oxidation, the absorption at 2939 and 2911 cm^−1^ reduced their intensity. No evidence of carbonyl from aldehydes was detected, but an intense peak appeared at 743 cm^−1^ (34). Figure 2B shows the Raman spectra of CHT-OHA blends, where peaks appeared at 2932, 2891, 1380, and 1102 cm^−1^ for the 1:1 ratio, and at 2934, 2890, 1374, and 1098 cm^−1^ for the 1:2 ratio. Bands below 1380 cm^−1^ showed low intensity, near 1098–1102 cm^−1^ assigned to ν_asym(C–O–C) and C–C stretching of HA [38]. In PEGDE-crosslinked samples, the bands at 1475–1468 cm^−1^ confirmed HA presence, and additional absorptions at 1112, 1123, and 844 cm^−1^ appeared with higher intensity in both CHT-OHA 1:1-PEGDE and 1:2-PEGDE formulations.

### 2.3. Thermal Behaviour by TGA

**Figure 3:**
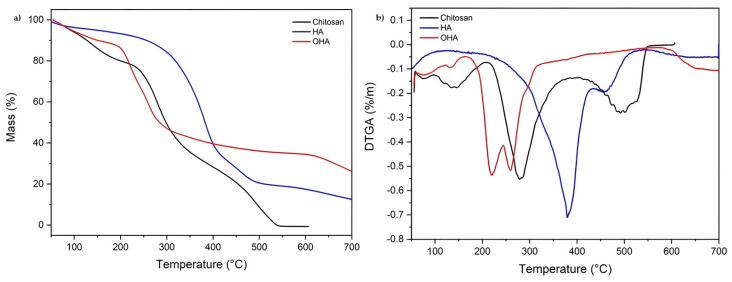

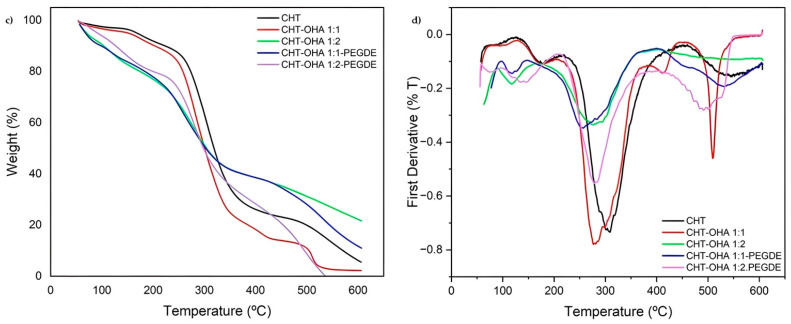
Thermogravimetric analysis (TGA) (**a**,**c**) and DTGA (**b**,**d**) thermograms of chitosan (CHT), hyaluronic acid (HA), oxidized hyaluronic acid (OHA), chitosan/oxidized hyaluronic acid (CHT-OHA) blends, and chitosan/oxidized hyaluronic acid (CHT-OHA) PEGDE-crosslinked sponges.

Figure 3 presents TGA and DTGA thermograms of chitosan (CHT), hyaluronic acid, and oxidized hyaluronic acid (Figure 3a–c) and blends of chitosan/oxidized hyaluronic acid (CHT-OHA 1:1), (CHT-OHA 1:2), blends of chitosan/oxidized hyaluronic acid crosslinked with polyethylene glycol diglycidyl ether (CHT-OHA 1:1-PEGDE), and (CHT-OHA 1:2-PEGDE) in Figure 3c,d.

The TGA thermograms clearly showed that HA (Td_1_ = 387 °C) was more thermally stable than CH (Td_1_ = 308 °C), but after oxidation, the HA thermal stability decreased, showing two decomposition temperatures at 222 and 262 °C. In agreement with this, CHT-OHA blends showed a reduced thermal stability independent of the extent of hyaluronic acid oxidation. Unexpectedly, PEGDE crosslinking did not increase their thermal stability as Td_1_ remained almost the same, except for CHT-OHA 1:2-PEGDE, where it significantly reduced Td_1_. A summary of the thermal properties and the temperature for 50% weight loss is shown in Table 1.

For all CHT-based sponges, weight loss began around 50 °C, as observed in Figure 3a,b. The weight change in this region may be attributed to the moisture loss from the samples, in accordance with the literature. According to Bwatanglang et al., strong degradation occurs between 250 °C and 350 °C for all samples containing chitosan, attributed to CO_2_ loss [39].

A detailed inspection of each thermogram revealed that the mass loss profile consists of three main stages; in Figure 3a, chitosan exhibits a mass loss of approximately 8% (175 °C) in the first stage, resulting from the removal of surface-bound water molecules, characteristic of chitosan due to water bound by hydrogen bonds with hydroxyl groups (–OH) (37). In the second stage, the sponge shows a 40% mass loss at 308 °C and an 85% mass loss at 550 °C. This mass loss can be attributed to the partial decomposition of the chitosan structure [40,41]. The pristine HA showed the loss of water at Td_1_ = 57 °C, a merged second transition Td_2_ with peaks at 333 and 387 °C, and the last decomposition Td_3_ at 461 °C. Periodate oxidation rendered decomposition at Td_1_ = 66 °C and 124 °C, Td_2_ = 225 °C and 262 °C and Td_3_ = 305 °C. The CHT-OHA 1:1 sample exhibited a thermogram similar to that of chitosan in the first two stages; however, in the third stage, the mass loss was approximately 93% at a temperature of 510 °C. Samples with the crosslinker PEGDE showed a 10% mass loss at 125 °C in the first stage, a 40% mass loss at 275 °C in the second stage, and finally, a third stage where 80% of its mass was lost at 500 °C.

The pure chitosan sponge exhibited the highest initial degradation temperature (308 °C) among the samples, while the CHT-OHA 1:1-PEGDE sponge showed the lowest (257 °C). However, the residual mass of CHT-OHA 1:2 and CHT-OHA 1:1-PEGDGE after degradation was higher, i.e., 22% and 11%, respectively, as shown in Table 1. The CHT-OHA 1:2 sponge exhibited the highest thermal stability (Td_1_ = 278 °C) among the crosslinked blends, and this behavior can be attributed to the partial crosslinking between chitosan and oxidized hyaluronic acid. This enhancement in thermal resistance suggests that the material possesses improved robustness under both thermal and physiological stress conditions. In this context, the higher decomposition temperature observed for CHT-OHA 1:2-PEGDE reflects greater molecular cohesion within the polymeric network, which may translate in vivo into a slower and more controlled degradation profile. Such stability is desirable for maintaining the scaffold’s mechanical integrity over time, supporting tissue regeneration, and ensuring the sustained release of bioactive molecules in the oral environment. Liu et al. assert that, regardless of temperature, the thermal stability of chitosan with OHA is lower than that of pure chitosan, as they indicate that when crosslinking agents are added to the mixture, the pH value decreases, forming intra- and intermolecular hydrogen bonds weakened by the abundance of ionic H+, making the structure more susceptible to thermal decomposition compared to pure chitosan [42]. The present findings indicate that when PEGDE is incorporated as a secondary crosslinker, the CHT-OHA network retains its thermal resistance. This suggests that the presence of PEGDE reinforces molecular cohesion through covalent bonds that stabilize the polymeric matrix, compensating for the potential weakening of hydrogen bonds reported by Liu et al. Consequently, the high decomposition temperature observed for the CHT-OHA 1:2-PEGDE formulation reflects a more compact and robust structure, which may translate in vivo into a slower and more controlled degradation profile. Such thermal and structural stability is desirable for maintaining the scaffold’s mechanical integrity over time, supporting tissue regeneration, and ensuring the sustained release of bioactive molecules within the oral environment. PEGDE incorporation promoted the formation of a more compact and cohesive polymer network that partially restricted chain mobility, delaying the onset of decomposition. Similar findings have been reported for PEGDE-crosslinked chitosan systems, where ether linkages between PEGDE and chitosan improved structural integrity and thermal resistance, despite producing only moderate increases in overall stability [18]. In this context, the improved decomposition behavior of the CHT-HA-PEGDE formulation can be attributed to the partial stabilization of the polymer chains through these covalent ether bonds, resulting in better performance under thermal or physiological stress conditions.

### 2.4. Morphology by SEM

**Figure 4:**
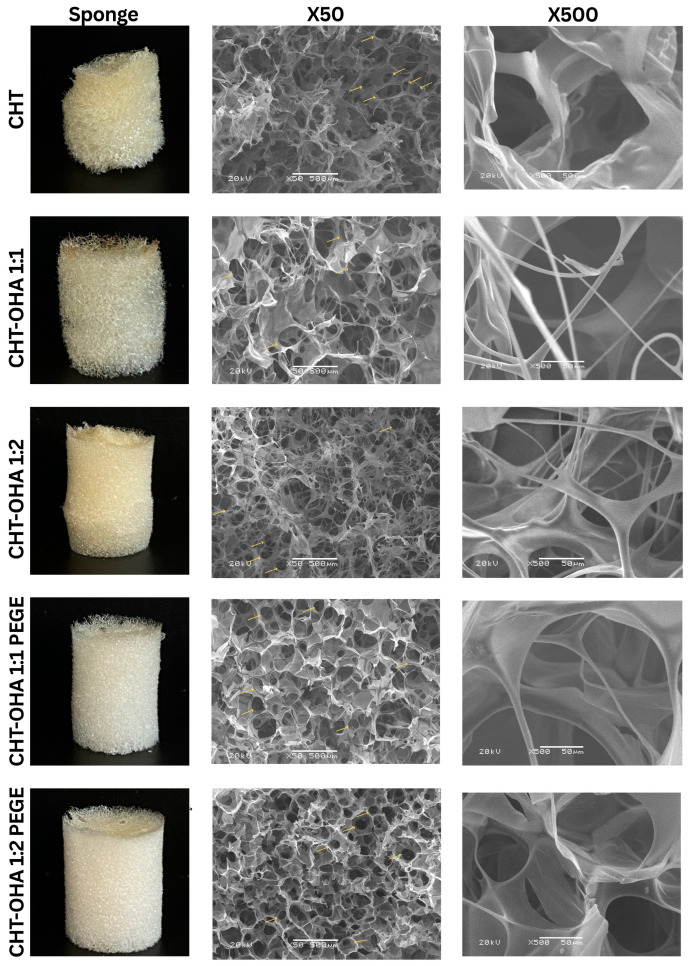
SEM surface morphology of CHT. Mixtures of chitosan/oxidized hyaluronic acid (CHT-OHA 1:1), (CHT-OHA 1:2); mixtures of chitosan/sodium hyaluronate/polyethylene glycol diglycidyl ether (CHT-OHA 1:1-PEGDE), and (CHT-OHA 1:2 PEGDE).

SEM analysis confirmed that all chitosan sponges subjected to different treatments (OHA and PEGDE) exhibited a three-dimensional network structure, highlighting an interconnected porous surface. The average theoretical pore diameter in chitosan and oxidized hyaluronic acid sponges ranged from ~97 to 231 μm (see Figure 4), encompassing the interval ~116 to 131 μm observed in sponges crosslinked with PEGDE. These quantitative estimations, derived from calibrated SEM micrographs and supported by experimental porosity data (92.8–95.0%), are consistent with previously reported values for chitosan–hyaluronic acid systems, confirming the robustness and reliability of the morphological analysis.

The variation in pore size may be attributed to the higher crosslinking density in the network of the samples, indicating a restriction of interactions within the polymeric matrix during the lyophilization process. This phenomenon could be responsible for forming smaller and non-uniform pores [43,44]. Previous studies have suggested that the presence of a porous structure in sponges can significantly contribute to good permeability, thereby promoting cell growth [43].

As evidenced in the images (Figure 4), the micrographs of chitosan and CHT-OHA samples displayed a morphology with large pores distributed heterogeneously. In contrast, the sponge of chitosan-oxidized hyaluronic acid (1:2) crosslinked with PEGDE exhibited a more homogeneous and dense morphology, characterized by smaller circular and uniform pores (Figure 4). According to Liu et al., the molar ratio of the crosslinking agent influences the pore size, as a higher ratio allows for the oxidation of more functional groups in the solution, facilitating the crosslinking with chitosan and forming a denser network [42].

The density of the samples was calculated experimentally in order to determine the percentage of porosity using the previously reported density of chitosan (0.3 g/cm^3^), following the method described elsewhere [45]. The sponges showed a density of 0.016, 0.015, 0.019, 0.021, and 0.021 g/cm^3^ for CHT, CHT-OHA 1:1, CHT-OHA 1:2, CHT-OHA 1:1-PEGDE, CHT-OHA 1:2-PEGDE, respectively, and a porosity of 93.7%, 95.0%, 93.5%, 93.0%, and 92.8% for CHT, CHT-OHA 1:1, CHT-OHA 1:2, CHT-OHA 1:1-PEGDE, CHT-OHA 1:2-PEGDE, respectively. Tan et al. indicate that the porous structure, whether large or small pores, is linked to the crosslinking density, which imparts a more closed network structure in biomaterials due to additional electrostatic crosslinking [46]. These findings align with the results obtained, as morphologies of smaller and more uniform pores are observed in sponges with a higher degree of HA oxidation and those containing PEGDE compared to pure chitosan.

The importance of the porous structure has been emphasized in the literature, as high porosity confirms the crosslinking of compounds. During the crosslinking process, the polymers form a three-dimensional network, resulting in spaces or pores within the structure. These pores are the direct result of the crosslinked polymer chains. In sponges formed from hyaluronic acid and chitosan, the presence of a porous structure means that the crosslinking agent PEGDE has effectively connected the polymer chains, creating a stable semi-interpenetrated network [28,47]. In turn, this property improves tissue adhesion, accelerates absorption, prolongs drug effects, and facilitates efficient gas and nutrient exchange, all of which are beneficial for tissue regeneration processes [48].

These observations confirm that the microstructural features revealed by SEM directly correlate with the expected in vivo performance of the sponges. Porosity and water absorption capacity are critical physicochemical parameters that determine a dressing’s ability to maintain a moist environment, regulate exudate, and promote cellular regeneration [49]. The CHT-OHA sponges exhibited high porosity values (92.8–95.0%), while the CHT-OHA 1:2-PEGDE formulation displayed a denser and more uniform structure with an optimal pore size distribution. This morphological configuration is particularly advantageous, as it supports tissue adhesion, nutrient diffusion, and vascular ingrowth (angiogenesis), all essential processes for wound healing in the oral cavity. Moreover, the presence of large, interconnected pores (>100 µm) is generally associated with enhanced cell migration and differentiation, thereby facilitating effective tissue integration and regeneration in vivo.

In this context, the structural and mechanical features of the PEGDE-crosslinked CHT-OHA sponges appear to complement each other. The CHT-OHA 1:2-PEGDE formulation not only exhibited a more homogeneous and compact architecture but also demonstrated the highest elastic modulus (17.2 ± 7.0 kPa) and compressive strength. Such a combination of high porosity and mechanical robustness may contribute to maintaining the integrity of the dressing under masticatory forces, sustaining a humid microenvironment, and favoring gradual degradation and tissue remodeling. Collectively, these characteristics are expected to provide an optimal framework for wound healing dynamics and tissue regeneration within the complex oral environment.

### 2.5. Compressive Mechanical Properties

Compression mechanical tests were conducted to determine the elastic modulus and compression stress (Table 2). The Young’s modulus (E) of the sponges was calculated between 10% and 15% deformation. The stress (σ 10%) of the sponges was calculated at maximum load in all samples. The results show that the material with the highest modulus is the scaffolds of chitosan crosslinked with oxidized hyaluronic acid at 1:2 and PEGDE.

The results show similarities with those reported by Maiz et al., who emphasized that the deformation required to reach a maximum of 70–80% depends directly on the material crosslinking, as detailed in Table 2. They also noted that a low CHT content and a high HA content in the scaffold facilitate deformation. In contrast, sponges with a high crosslinking density require higher efforts to deform and simultaneously exhibit higher elastic moduli, suggesting a directly proportional relationship [44].

These findings align with the density results of the samples, where the denser and more deformation-resistant sponge has higher crosslinking (CHT-OHA 1:2-PEGDE). One-Way ANOVA (*p*-value < 0.1) and an ANOVA with Tukey’s test (*p*-value < 0.05) showed statistical significance regarding elastic modulus and maximum compression stresses, calculated at a 10–15% deformation in the sponges.

In addition to the compressive stress at 10% strain (σ 10%), the mechanical analysis also included the determination of Young’s modulus (E), which provides complementary information on the stiffness and elastic behavior of the sponges. The CHT-OHA 1:2-PEGDE formulation presented the highest Young’s modulus (17.2 ± 7.0 kPa) and compressive stress at 10% strain (2.0 ± 0.6 kPa), compared to pure chitosan (11.2 ± 1.5 kPa, 1.5 ± 0.3 kPa) and CHT-OHA 1:2 (11.1 ± 3.8 kPa, 1.3 ± 0.3 kPa). These findings demonstrate that the incorporation of OHA alone did not significantly alter the stiffness, whereas the combined effect of OHA and PEGDE produced a denser polymer network with higher stiffness and greater resistance to deformation. This improvement correlates with the higher density of the crosslinked sponges (0.021 g/cm^3^ vs. 0.016 g/cm^3^ for pure chitosan) and SEM observations, which show smaller and more uniform pores. While the present study focused on the elastic region up to a 15% strain, mechanical trends suggest that the CHT-OHA 1:2-PEGDE sponge would better withstand higher compressive loads due to its higher crosslink density. This behavior is consistent with reports on similar chitosan–OHA systems, where higher crosslinking and polymer concentration improved viscoelastic performance and mechanical stability under deformation [50,51]. Future studies will include cyclic and viscoelastic testing to better elucidate the mechanical response of these scaffolds under conditions that mimic the dynamic oral environment.

Collectively, our findings reveal that the mechanical properties of the PEGDE-crosslinked CHT-OHA sponges are closely related to their potential in vivo behavior in the oral environment. In particular, the CHT-OHA 1:2-PEGDE formulation exhibited the highest elastic modulus (17.2 ± 7.0 kPa) and compressive strength, indicating a denser crosslinking network. This increased rigidity and resistance to deformation are expected to enhance structural stability under masticatory forces, provide sustained tissue support, and favor optimal integration with the oral mucosa [52]. Moreover, a higher crosslinking density may contribute to controlled degradation and improved moisture retention, both of which are desirable for maintaining a moist wound environment and promoting effective healing kinetics in the oral cavity.

### 2.6. Evaluation of Cell Viability

**Figure 5:**
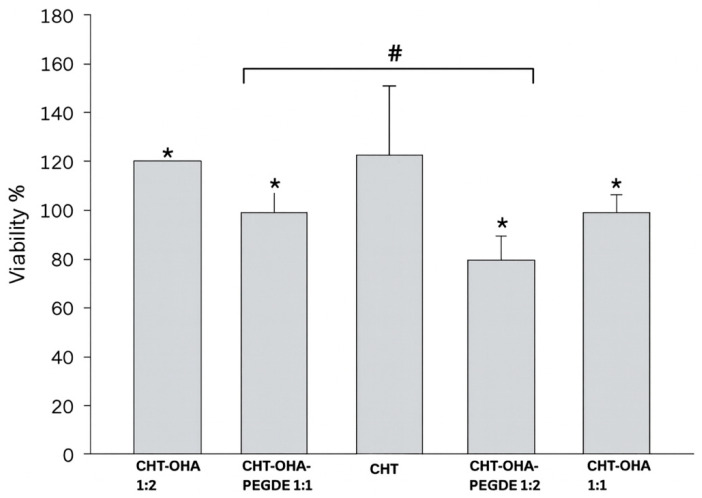
Viability of fibroblasts in direct contact with chitosan/oxidized hyaluronic acid (CHT-OHA 1:1), (CHT-OHA 1:2), and mixtures of chitosan/sodium hyaluronate/polyethylene glycol diglycidyl ether (CHT-OHA-PEGDE 1:1) and (CHT-OHA-PEGDE 1:2). Values represent mean ± SD (*n* = 3). Asterisks (*) indicate significant differences (*p* < 0.05) compared to the control (CHT). # = significant difference between groups (*p* < 0.05).

Cytotoxic effects can hinder the biomaterial’s integration by modifying the natural incorporation process [44]. This necessitates the assessment of the biomaterial’s cytotoxicity. Samples containing OHA (CHT-OHA 1:1 and CHT-OHA 1:2) exhibited higher cell proliferation, demonstrating the beneficial effect of incorporating OHA into the chitosan biomaterial (Figure 5, Table 3).

According to ISO 10993-5:1999 [53], materials with cell viability above 75% are considered non-cytotoxic. All tested formulations exceeded this threshold, confirming the biocompatibility of the developed sponges. A significant difference (*p* < 0.05) was detected only between the CHT-OHA and CHT-OHA-PEGDE groups, possibly due to the presence of residual reactive PEGDE groups.

Nguyen et al. explored the oxidation degree of AHO for hydrogel formation, investigating the impact of different oxidation degrees and volume ratios of chitosan and hyaluronic acid. They reported that a lower degree of OHA oxidation favored cell proliferation, cell adhesion, and wound healing in both in vitro and in vivo studies [46]. Khorshidi et al. synthesized a hydrogel with varying concentrations of oxidized constituents (oxidized alginate and hyaluronic acid) and gelatin. They demonstrated that, regardless of composition, the hydrogels preserved cell viability and proliferation in mesenchymal stem cells [48]. Weng et al. developed self-crosslinking hydrogels formulated with OHA and the amino group of gelatin in the presence of borax. When tested with dermal fibroblasts, these hydrogels improved mechanical properties and biocompatibility [54].

All studied samples (chitosan, chitosan–oxidized hyaluronic acid, and chitosan–oxidized hyaluronic acid-PEGDE) demonstrated cell viability higher than that of the positive control, confirming the non-cytotoxic nature of the created sponges and making them suitable for oral ulcer treatment [48,54].

This result is supported by ANOVA statistical analysis with Tukey’s test, yielding a *p*-value of 0.05, which is statistically significant for all sponges. A statistically significant difference was only detected between CHT-OHA and CHT-OHA-PEGDE sponges. This effect could be attributed to PEGDE, as residual crosslinking molecules not bound to other molecules might be toxic [55]. The toxicity of PEGDE can be attributed to its two terminal epoxy groups, which are considered to have high reactivity [54]. The epoxy rings of PEGDE would interact with the hydroxyl groups of HA, forming ether-type bonds [56]. Kim et al. reported cytotoxicity of high concentrations of PEGDE (>100 ppm) in human keratinocytes and dermal fibroblasts, breaking the cell membrane and generating intracellular reactive oxygen species [35]. However, the PEGDE used in this study had a molecular weight of 500 Mn, which is considered non-cytotoxic, as evidenced by the results. Chitosan stimulates fibroblast proliferation, and this effect has been attributed to the degree of deacetylation, which correlates positively with fibroblast activation [56,57]. All chitosan sponges, except those with PEGDE crosslinker, exhibited favorable growth patterns after two days of incubation compared to the control group, suggesting that the manufactured biomaterials have no cytotoxic effect against fibroblasts. Similar to our results, it has been reported that the highest cell proliferation was observed in the sponge composed of chitosan and oxidized hyaluronic acid in a 1:2 ratio, followed by pure chitosan and chitosan and oxidized hyaluronic acid in a 1:1 ratio [57]. Cell viabilities for all samples are near or above 100%, indicating excellent cell compatibility and low toxicity of the sponges. Studies have indicated that when the cell viability value exceeds 100%, the studied biomaterials or samples can promote cell differentiation and growth [58].

Several biomaterials have been explored for the management of oral lesions, including collagen and alginate-based systems; however, chitosan (CS) and its derivatives have gained increasing attention due to their low cost, biocompatibility, biodegradability, and intrinsic antibacterial and anti-inflammatory properties. For example, Ryu et al. (2024) developed a trimethyl chitosan (TMC) hydrogel combined with human mesenchymal stem cells for the treatment of oral ulcers, achieving faster healing and greater cell retention in vivo [59]. Similarly, Shao and Zhou (2020) evaluated a mucoadhesive chitosan film for recurrent aphthous stomatitis, reporting a significant reduction in lesion size [27]. Chitosan has also been used in solution or biogel form, showing potential to reduce pain and improve healing of oral ulcers, although with limited evidence compared to corticosteroid-based treatments [60]. In this context, our study introduces a topical CHT/OHA sponge that represents an advancement over conventional chitosan-based systems by enhancing the scaffold’s mechanical strength and stability through crosslinking with oxidized hyaluronic acid (OHA). Unlike films or gels, this porous and mechanically robust sponge structure is designed to sustain tissue support, promote integration with the oral mucosa, and provide a more stable platform for wound healing in the dynamic and humid oral environment [61].

Alginate-based hydrogels and topical formulations, such as Faringel^®^, have also demonstrated efficacy in pain reduction and wound protection. However, compared with these injectable or gel-based systems, the CHT/OHA sponge developed in our study provides a solid, mechanically stable, and highly porous scaffold. This configuration offers improved compressive strength and tissue integration, representing a promising alternative to conventional HA or collagen-based materials for oral mucosal lesion management [62].

Taken together, these comparisons highlight that while numerous natural biomaterials, such as collagen, alginate, and hyaluronic acid, have demonstrated potential for the treatment of oral wounds, their limitations in terms of mechanical stability and antibacterial performance remain evident. The PEGDE-crosslinked CHT-OHA sponge developed in this study addresses these challenges by combining the regenerative and biocompatible properties of hyaluronic acid with the structural robustness and antimicrobial activity of chitosan. Therefore, this formulation represents a promising basis for the next generation of bioactive scaffolds designed to promote controlled degradation, tissue integration, and sustained healing in the complex oral environment.

However, to fully corroborate these advantages and move toward clinical application, further validation is essential. Future work should include in vivo validation to confirm the biological performance of the CHT/OHA sponges under physiological conditions. Although our study focused on in vitro physicochemical and cytocompatibility assessments, these results must be corroborated in animal models to better simulate the dynamic and humid oral environment. Similar studies using chitosan- and hyaluronic acid-based hydrogels have shown accelerated healing in in vivo models, supporting this direction. Such validation will be crucial to evaluate long-term stability, degradation behavior, and tissue integration, ultimately bridging the gap between laboratory findings and clinical translation.

## 3. Materials and Methods

### 3.1. Materials Selection

Chitosan (Mn = 183.364 g/mol, average molecular weight, deacetylation > 75%, lot STBF3282V) was supplied by Sigma-Aldrich (St. Louis, MO, USA). Acetic acid (99.7% purity) was obtained from J.T. Baker (Radnor, PA, USA). Oral grade low molecular weight sodium hyaluronate (≥98%) was purchased from Bioibérica S.A.U. (Barcelona, Spain). Sodium periodate (ACS grade, ≥99.8%) and polyethylene glycol diglycidyl ether (average Mn 500) were obtained from Sigma-Aldrich (St. Louis, MO, USA).

Human neonatal dermal fibroblasts (ATCC PCS-201-010, lot no. 64154600) were used for cytocompatibility assessment.

Cells were maintained in Biowest DMEM low-glucose medium (Biowest, Nuaillé, France) supplemented with 10% fetal bovine serum and 1% antibiotic–antimycotic mixture (penicillin/streptomycin/amphotericin B). All cell culture reagents were purchased from Biowest (Nuaillé, France).

### 3.2. Methods

#### 3.2.1. Preparation of Uncrosslinked Chitosan Sponges

Chitosan sponges (CHT) were prepared by dissolving 100 mg of chitosan in 40 mL of 0.7 M acetic acid with constant stirring until complete solubilization was achieved. This concentration (0.25% *w*/*v*) was selected to provide sufficient viscosity for homogeneous mixing with oxidized hyaluronic acid (OHA) solutions while maintaining adequate pore-forming capacity during lyophilization. Although higher concentrations of chitosan (1–2%) are frequently reported, lower concentrations have been successfully used to obtain stable porous matrices when mixed with secondary polysaccharides, such as OHA, improving the structural integrity of the resulting scaffold. The solutions were poured into molds and frozen at −20 °C for 72 h, followed by lyophilization for 5 days to obtain the final sponges with an interconnected porous network.

#### 3.2.2. Preparation of Chitosan and Oxidized Hyaluronic Acid (OHA) Sponges in 1:1 and 1:2 Ratios

Oxidized hyaluronic acid (OHA) was prepared using a modified periodate oxidation method. Briefly, 1 g of sodium hyaluronate (HA) was dissolved in 100 mL of distilled water with constant stirring until a homogeneous solution was obtained. Sodium periodate (NaIO_4_) was then added in molar ratios of 1:1 and 1:2 (NaIO_4_:HA repeating unit) to achieve two levels of oxidation. The reaction mixture was kept under continuous stirring in the dark at 25 °C for 12 h to prevent overoxidation or light-induced degradation. The reaction was quenched by adding ethylene glycol (0.5 mL) and stirring for an additional 30 min. The oxidized product was dialyzed using a membrane with a molecular weight cutoff (MWCO) of 3500 Da against distilled water for 72 h, with frequent water changes, to remove unreacted reactants and byproducts. The purified solution was subsequently frozen and lyophilized to obtain oxidized hyaluronic acid (OHA), which was stored at 4 °C until further use. Solutions of OHA with chitosan solution were prepared, and the corresponding sponge was obtained as described before.

**Figure 6:**
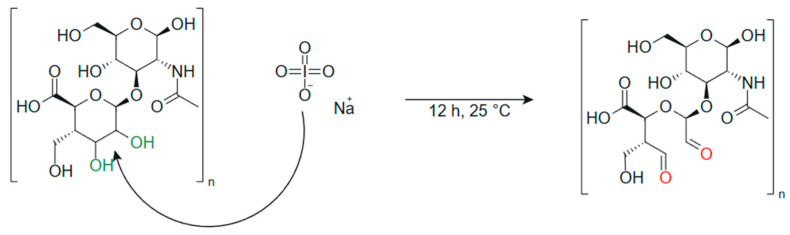
Oxidation of hyaluronic acid (HA) with sodium periodate to produce oxidized hyaluronic acid (OHA) containing aldehyde groups. Hydroxyl groups involved in the oxidation reaction are highlighted in green, and the newly formed aldehyde groups are shown in red.

The oxidation ratios (1:1 and 1:2) were selected to modulate the degree of oxidation and control the amount of aldehyde groups generated on the HA backbone, which directly affects the crosslinking density and mechanical strength of the resulting CHT-OHA sponges [29]. A higher NaIO_4_:HA ratio (1:1) was expected to produce a higher number of reactive aldehyde groups, which would enhance the formation of Schiff base bonds with chitosan and promote the development of a denser and more stable three-dimensional polymer network. A schematic of the reaction pathway illustrating the oxidation of HA with NaIO_4_ and the generation of aldehyde groups is provided in Figure 6 to clarify the chemical transformation before crosslinking with chitosan.

#### 3.2.3. Preparation of PEGDE-Crosslinked Chitosan/Oxidized Hyaluronic Acid (OHA) Sponges

Considering the high-water solubility of HA and the limited crosslinking reaction between aldehyde groups in OHA and amino groups in the chitosan to form imines, these polymers were further crosslinked with PEGDE as previously reported [28].

The previously described methodology was performed by pouring 25 mL of chitosan solution, 25 mL of OHA, at either a 1:1 or 1:2 oxidation ratio, and 25 mL of PEGDE. Solutions were placed on the stirring plate for 1 h.

**Figure 7:**
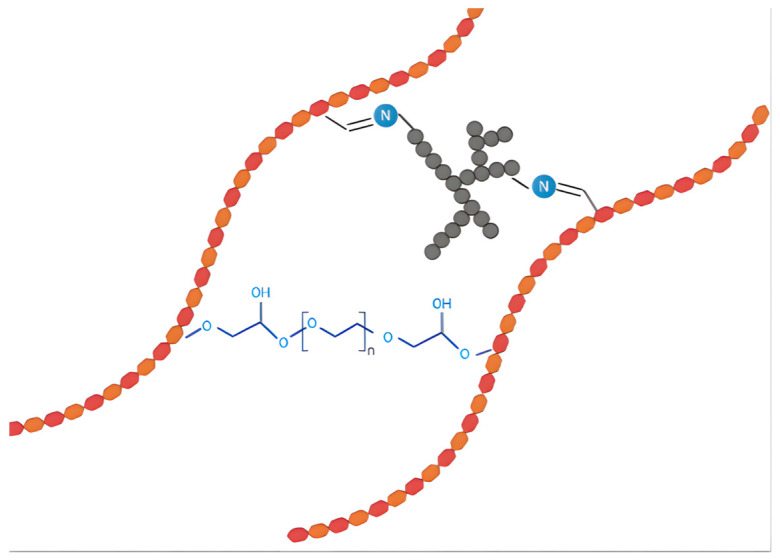
Crosslinking mechanism between chitosan (CHT), oxidized hyaluronic acid (OHA), and PEGDE, showing the formation of imine and covalent bonds within the three-dimensional polymeric network.

Finally, the mixtures were poured into 48-well cell culture plates, with each well containing 1.2 mL of the solution, and these were frozen (−20 °C) for 72 h to render a double crosslinked network (Figure 7). They were lyophilized for 5 days to obtain sponges using a Labconco FreeZone Benchtop lyophilizer (Labconco Corporation, Kansas City, MO, USA). Subsequently, physicochemical and mechanical characterization was carried out, including SEM, TGA, FTIR, Raman spectroscopy, and compression mechanical studies.

### 3.3. Composition and Structural Characterization of Crosslinked Sponges

#### 3.3.1. Fourier Transform Infrared Spectroscopy (FTIR)

The FTIR spectra were obtained from chitosan and modified chitosan sponges using a Nicolet 8700 spectrophotometer from Thermo Scientific (Madison, WI, USA), in an attenuated total reflectance (ATR) system, utilizing a zinc selenide crystal (ZnSe). The spectra were acquired as an average of 100 scans in the range of 4000 to 650 cm^−1^, with a resolution of 4 cm^−1^, and were reported in transmittance mode.

#### 3.3.2. Raman Spectroscopy

Raman spectra were obtained using an InVia™ Raman microscope from Renishaw (Wotton-under-Edge, Gloucestershire, UK). A laser with a wavelength of 633 nm was employed in the spectral range of 100–3200 cm^−1^, applying 100% power, with exposure times ranging from 10 to 60 s and bleaching time (fluorescence removal) between 0 and 60 s. Both time parameters varied according to the sample’s response.

#### 3.3.3. Thermogravimetric Analysis (TGA)

Thermogravimetric analysis was performed using a TGA-7 thermogravimetric analyzer from Perkin Elmer (Waltham, MA, USA), in a temperature range of 50 to 600 °C with a heating rate of 10 °C/min under a nitrogen (N2) atmosphere.

#### 3.3.4. Scanning Electron Microscopy (SEM)

The morphology and pore size of the sponges were observed using a scanning electron microscope, JEOL JSM-6360 LV (Akishima, Tokyo, Japan), operating at 20 kV. Samples were analyzed at magnifications of 50 and 500×. For microscope observation, the sponges were initially coated with a thin layer of gold using a Denton Vacuum Desk-II sputter coater (Moorestown, NJ, USA) for 1 min. To determine porosity, calculations were taken from 5 samples in each image and analyzed with the “J” image processor. Diameter calculated from the following equation:

$$D_{theo}={\left(\frac{4A_{cal}}{\pi }\right)}^{\frac{1}{2}}$$

where $D_{theo}$ and $A_{cal}$ are the theoretical diameter and the calculated area, respectively.

#### 3.3.5. Mechanical Analysis

The specimens used (*n* = 5) were dry foam samples with a height of 5 mm (±0.3 mm) and a diameter of 4 mm (±0.2 mm). The tests were carried out using a Shimadzu AGS-X universal testing machine (Shimadzu Corporation, Kyoto, Japan) at 25 °C, with a 100 N load cell and a crosshead speed of 1 mm/min. Compression strength (σ, MPa) was calculated using the following equation:

$$\sigma =\frac{4F}{\pi D^{2}}$$

where *F* is the maximum load applied (N), and *D* is the diameter of the cylinder (mm). The following parameters are reported: modulus at 10–15% deformation, stress at 10% deformation (*σ* 10%), maximum strength, and maximum deformation when the curve becomes asymptotic, and the test is stopped to prevent the platens from coming into contact. An ANOVA with Tukey’s test was performed to determine differences between study groups for the elastic modulus and maximum compressive stresses of the materials. The modulus was calculated at 10–15% strain in sponges. The difference was considered statistically significant at *p* < 0.05.

#### 3.3.6. Biocompatibility Studies

##### Cell Viability and Proliferation Studies

Cell adhesion and proliferation were assessed using neonatal dermal fibroblasts (ATCC) through the MTS CellTiter 96^®^ assay under indirect contact conditions. A non-radioactive cell proliferation assay was performed on different samples in 96-well culture plates. A positive control (C+), consisting of cells with only culture medium, and a negative or death control (C−) based on hydrogen peroxide, was included. For each sample, 5 × 10^4^ cells were seeded in a volume of 100 μL and incubated for 24 h at 37 °C with 95% humidity and 5% CO_2_ atmosphere.

Subsequently, the medium was replaced with culture medium containing extracts (eluates) obtained from the sponges. Extracts were obtained using 2.5 mg/mL of medium enriched with 10% FBS and 1% antibiotic. The medium with the sponge was centrifuged at 2500 rpm for 5 min. The cells were conditioned for 24 h. After this time, the culture medium was removed, replaced with 100 μL of each of the eluates, and incubated for an additional 48 h. After this time, the MTS working solution was prepared according to the manufacturer’s instructions, and 20 μL of this solution was added to each sample and incubated for 4 h under the same conditions mentioned earlier.

Finally, the samples were analyzed using a microplate reader (Thermo Scientific FC Multiskan^®^, Vantaa, Finland) at 493 nm, and the absorbance values were compared with the control sample. The absorbance values are directly proportional to the number of living cells in culture. This result is verified by ANOVA statistical analysis with Tukey’s test, yielding a value of *p* = 0.05, which was considered statistically significant for all sponges.

### 3.4. Statistical Analysis

All experiments were carried out in triplicate, and the results are presented as mean ± standard deviation (SD). Statistical differences among groups were analyzed by one-way analysis of variance (ANOVA) followed by Tukey’s post hoc test, using Minitab 24.1 (Minitab LLC, State College, PA, USA) and GraphPad Prism 9.0 (GraphPad Software, La Jolla, CA, USA). A *p*-value < 0.05 was considered statistically significant.

## 4. Conclusions

This study successfully developed and characterized chitosan/oxidized hyaluronic acid (CHT-OHA) sponges as potential biomaterials for the treatment of oral mucosal lesions. The results demonstrated that the incorporation of oxidized hyaluronic acid (OHA) and crosslinking with PEGDE significantly improved the physicochemical and mechanical properties of the sponges, generating a more stable, elastic, and structurally cohesive network. The CHT-OHA 1:2-PEGDE formulation exhibited the highest elastic modulus and compressive strength, indicating greater resistance to deformation and higher crosslinking density.

From a biological perspective, the sponges displayed adequate in vitro biocompatibility, with no cytotoxic effects, suggesting their potential as scaffolds that could promote cell adhesion and proliferation in oral soft tissue repair. The porous architecture and hydrophilic nature of the materials meet the requirements of wound healing environments. However, these findings represent preliminary in vitro evidence, and the clinical application of these materials requires further in vivo validation. Future studies should investigate their degradation profile, biointegration capacity, and wound healing ability in animal models, as well as their long-term biocompatibility and functional stability under dynamic oral conditions.

## Figures and Tables

**Table 1:** Maximum decomposition temperatures and weight loss of CHT-based sponges.

Sponges	Td (°C)	T (°C) at 50% of Weight Loss	% Residual Mass at 600 °C
CHT	308	315	6
CHT-OHA 1:1	278	302	2
CHT-OHA 1:2	275	304	22
CHT-OHA 1:1-PEGDE	257	305	11
CHT-OHA 1:2-PEGDE	278	300	0

**Table 2:** Mechanical properties of scaffolds under compression.

Scaffolds	Young’s Modulus (*E*) kPa	Compressive Stress (σ 10%) kPa
CHT	11.2 ± 1.5	1.5 ± 0.3
CHT-OHA 1:1	5.9 ± 1.1	0.9 ± 0.1
CHT-OHA 1:2	11.1± 3.8	1.3 ± 0.3
CHT-OHA 1:1-PEGDE	7.7 ± 2.9	1.5 ± 0.2
CHT-OHA 1:2-PEGDE	17.2 ± 7.0	2.0 ± 0.6

**Table 3:** Cell viability (%) of fibroblasts cultured with chitosan and chitosan/oxidized hyaluronic acid sponges. Mean ± SD, *n* = 3. Statistical analysis performed by ANOVA followed by Tukey’s test (*p* < 0.05).

Sample ID	Composition	Cell Viability (%)	Statistical Significance (vs. Control)	Interpretation
CHT-OHA 1:2	Chitosan + Oxidized Hyaluronic Acid (1:2)	120 ± 6	n.s.	Highest proliferation; non-cytotoxic
CHT-OHA-PEGDE 1:1	Chitosan + OHA + PEGDE (1:1)	96 ± 7	n.s.	Slight reduction; non-cytotoxic
CHT (Pure)	Chitosan	118 ± 15	n.s.	High cell viability; supports fibroblast growth
CHT-OHA-PEGDE 1:2	Chitosan + OHA + PEGDE (1:2)	74 ± 9	*p* < 0.05	Slightly lower viability; within ISO 10993-5 range
CHT-OHA 1:1	Chitosan + Oxidized Hyaluronic Acid (1:1)	95 ± 8	n.s.	Good compatibility; non-cytotoxic

n.s. = not significant.

## Data Availability

The data presented in this study are available upon request from the corresponding author. The data are not publicly available as the content was derived from a thesis for a Bachelor’s degree in dentistry.
